# Susceptibility of Fluconazole-Resistant *Candida albicans* to Thyme Essential Oil

**DOI:** 10.3390/microorganisms9122454

**Published:** 2021-11-28

**Authors:** Najla A Alshaikh, Kahkashan Perveen

**Affiliations:** Department of Botany and Microbiology, College of Science, King Saud University, Riyadh 11495, Saudi Arabia; nalshaikh@ksu.edu.sa

**Keywords:** *Candida albicans*, essential oil, thyme, fluconazole resistance

## Abstract

*Candida* spp. is the most common microbial pathogen in fungal infections. There has been a tremendous increase in cases of candidiasis, especially among critically ill non-neutropenic patients. *Candida albicans*’ isolates were procured from the Prince Sultan Military Hospital, Riyadh, KSA. The isolates were characterized for their identification using CHROMagar, carbohydrate metabolism, germ tube formation, and RAPD-PCR techniques. The essential oil of *Thymus vulgaris* was obtained by hydro-distillation and characterized to decipher the major bioactive phytoconstituents. The antifungal activity of the thyme essential oil (TEO) was evaluated against fluconazole-resistant *C. albicans* isolates. The major phytocomponents identified by GC/MS were thymol (68.1%) followed by γ-terpinene (8.9%), cymol (7.7%), caryophyllene (1.1%), linalool (1.4%). The TEO successfully reduced the growth of *C. albicans* isolates. At very low doses, the TEO proved to be fungi static and fungicidal. TEO also effectively inhibited the germ tube formation and budging of fungal pathogens. The time kill assays have shown that TEO was more effective against drug resistant clinical isolates than fluconazole. This study provides an array of experimental evidence regarding the therapeutic efficacy of TEO against the drug-resistant clinical isolates of *C. albicans*. The findings may be used in the development of a new antifungal agent accordingly.

## 1. Introduction

Among fungal commensal pathogens, *Candida* spp. is one of the major causative agents for human infections. It colonizes the mucosal surfaces of oral-pharyngeal, gastrointestinal, and urogenital tracts [1,2]. In the last few decades, there has been an overwhelming increase in the Candidiasis caused by *Candida* species. Out of different *Candida* species, *C. albicans* is the most prominent pathogen associated with serious fungal infection as it accounts for roughly 90% of the total cases [3,4]. Candidiasis is known as the most common invasive fungal infection in critically ill non-neutropenic patients [5]. Based on numerous studies conducted in the last decade for candidemia at various hospitals in Riyadh, it is obvious that *C. albicans* is the most prevalent species for such infections [1]. Moreover, other findings have also shown that *C. albicans* is the major species that causes vaginitis in Saudi Arabian women [6].

The irresponsible and abusive usage antimicrobials both in clinical and environmental settings has led to the development of the global spread of drug resistance among microbial pathogens. This has drastically reduced the therapeutic effectiveness of antimicrobial drugs and warranted a need for the search of alternative therapies to combat such infections. Essential oils have been shown to have excellent antifungal and antibacterial properties among natural products [7,8,9,10]. Many studies conducted on essential oils and natural extracts have proved that many natural products exhibit highly momentous antibiotic properties [11,12,13]. Essential oils derived from aromatic plants are well-known in traditional medicine as antimicrobial agents and are known for broad-spectrum activity, such as antifungal properties, food preservatives, inhibitors of mycotoxin production, antimycotic agents, etc. [14,15]. Owing to the excellent bioactivities, researchers have focused on the screening and identification of new chemical entities from natural products exhibiting excellent antimicrobial properties [16]. The known important classes of bioactive compounds for drug discovery are alkaloids, tannins, and phenolic compounds, etc. [17].

The essential oil of thyme (*Thymus* spp.) has antiseptic, bronchiolytic, antispasmodic, and antimicrobial qualities, making it a popular medical plant and food preservative [18]. Thyme’s medicinal potential is based on its flavonoids, thymol, eugenol, aliphatic phenols, as well as saponins, luteolin, and tetramethoxylated flavones [19]. Several investigations have indicated that thyme essential oil has antimicrobial properties, with phenols content being the most active. Thymus oils containing thymol and carvacrol have been of significant interest for some time due to the limited occurrence of these phenols in nature [13,20] Kowalczyk [21] recently stated that widespread use of thymol and thyme essential oil in the healthcare industry is highly promising, but that more study and analysis is needed. In this study, thyme essential oil (TEO) was isolated using hydro-distillation process and then tested against the clinical drug resistant *C. albicans* spp. isolates.

## 2. Materials and Methods

### 2.1. Collection and Characterization of Candida albicans

A total of 120 *Candida* species were procured from Prince Sultan Military Hospital, Riyadh, KSA. Based on the information about the source of isolation, the *Candida* isolates were divided into two groups: isolates from blood and isolates from vaginal culture. The fungal isolates were preliminary identified on the basis of microscopic and macroscopic characteristics by culturing on Sabouraud Dextrose Agar (SDA) medium (Merck, Darmstadt, Germany). These isolates were characterized based on CHROMagar, germ tube development, carbohydrate metabolism, and RAPD-PCR technique.

#### 2.1.1. Identification of *Candida* sp. by CHROMagar

The preliminary identification of yeast was done by growing the cultures on differential isolation CHROMagar medium (Paris, France). This growth medium facilitates the presumptive identification of clinical *Candida* species [22].

#### 2.1.2. Germ Tube Test for the Identification of *Candida albicans*

The isolated colonies were inoculated in horse serum and then incubated at 37 °C. Wet mount was prepared from inoculated horse serum and examined microscopically for production of germ tubes after 2–3 h of incubation [23].

#### 2.1.3. API 20c Aux System for *C. albicans* Identification

The strains of *C. albicans* were further identified by commercially available API 20c aux System for yeasts (BioMerieux, Marcy L’Etoile, France). Samples were prepared and loaded into microtubes as per the instruction of the manufacturer, followed by 48–72 h incubation at 29 °C. Turbidity more than the control cupel indicated a positive result [24].

#### 2.1.4. Differentiation between *C. albicans* Isolates by RAPD-PCR Technique

DNA from *Candida* samples were extracted using standard procedure [25]. The molecular typing of *Candida* spp. was carried out by RAPD-PCR using Ready-To-Go/RAPD analysis beads kit (GE Healthcare, Manufacturer, UK). *C. albicans* (ATCC 10231) was taken as positive control. The details of primers used is listed below. The amplification reaction was performed in a final volume of 25 μL containing 1 μL extracted genomic DNA (about 20 ng), 25 pmol single RAPD primer, and distilled water. The contents were mixed gently by vortexing. PCR was performed, carried out in a PTC0200 thermal cycler (Bio-Rad, Hercules, CA, USA). The temperature profile was: 1 cycle of 5 min at 95 °C, followed by 45 cycles of 1 min at 95 °C, 1 min at 36 °C, and 2 min at 72 °C. The amplification products were loaded onto 2% agarose gel and run in TBE buffer (90 mM boric acid, 90 mM Tris, and 2 mM EDTA, pH 8.3) at 120 V for 2.5 h. The products were detected by staining with ethidium bromide (0.5 μg/mL) and then visualized under UV light and photographed (Molecular Imager Gel Doc, Bio-Rad, Hercules, CA, USA). The following primers were used:

RSD11-(5′GCATATCAATAAGCGGAGGAAAAG-3′), OPG 14-(5′-GGATGAGACC-3′), RSD12-(5′ GGTCCGTGTTTCAAGACG-3′), and OPG 17-(5′-ACGACCGACA-3′).

#### 2.1.5. Fluconazole Susceptibility Test

The susceptibility of *C. albicans* were tested by E-test as instructed by the manufacturer (AB Biodisk North America Inc., Rodlphe St Durham, NC, USA). For the determination of minimum inhibitory concentration (MIC) of fluconazole, the fungal strains were grown overnight in the presence of varying concentrations (0–128 µg/mL) of fluconazole in Sabouraud Dextrose Broth (SDB) medium ((Merck, Darmstadt, Germany). and checked for visible growth (turbidity). The growth was further verified by spotting the cultures from broth onto Sabouraud agar plates.

### 2.2. Plant Material Collection and Extraction of Thyme Essential Oil (TEO)

The whole aerial part of the Thyme plant (*Thymus vulgaris*) was collected from a local market in Riyadh, Saudi Arabia. The identity of the plant was confirmed by the taxonomist, Prof. Najat Bukhari, Department of Botany and Microbiology, King Saud University with the voucher (P/M/014). Selection of the medicinal plant was based on ethnopharmacological (traditional) usage for the treatment of diseases in Saudi Arabia.

The extraction of essential oil was performed by adding 250 g of the aerial plant part in 200 mL distilled water and heating for 3 h at 100 °C using a Clevenger type apparatus [26]. The TEO vapours were condensed at 8 °C and collected in glass bottles. The TEO was then dried over anhydrous sodium sulphate and filtered. The TEO was stored at 4 °C in sealed brown vials until use. In this study, 2 kg plant material was used for extraction.

### 2.3. Gas Chromatography/Mass Spectrometry (GC/MS) Analysis of Thyme Essential Oil (TEO)

The phytochemical components of TEO was identified using Perkin Elmer (Clarus 500, Walthman, MA, USA) gas chromatograph (GC) equipped with flame ionization detector (FID) and DB-5 capillary silica column. The initial oven temperature was set to 40 °C for 1.08 min and then increased to 240 °C at the rate of 3 °C/min. The details of operating conditions are as follows: carrier gas; helium with a flow rate of 1.0 mL/min; injector and detector temperature: 250 and 300 °C, respectively; split ratio: 1:20. The interface temperature was 280 °C. The mass range (*m*/*z*) of recorded spectra was 35–375 amu. The mass spectra were taken at 70 Ev, whereas a thermo quest 2000 GC coupled with thermo fining, mass system and a DB-5 capillary column (30 m × 0.25 mm; 0.25 μm film thickness) was used. The rest of the operating systems were the same as for GC analysis. The compounds of the TEO were identified by comparing the hits of mass spectra with the MS computer library (NIST). The retention indices were calculated using a homologous series of n-alkanes (C6-C28).

### 2.4. Antifungal Activity of Thyme Essential Oil (TEO) against C. albicans Isolates

The TEO was tested for its efficacy against the drug resistant isolates of *C. albicans*.

#### 2.4.1. Inhibitory Effect of TEO on *C. albicans* Using Disc Diffusion Assay

The preliminary anticandidal activity of TEO was tested at varying oil concentrations (100, 30, 25, 20, 15, 10, 5, 3, and 2% *v*/*v*) against 20 isolates of *C. albicans* [27]. The TEO were reconstituted in 1% Tween-20 to enhance the oil solubility. A 100 µL inoculum was taken from the log phase and spread on SDA plates. Sterile discs of 5 mm diameter were loaded with 10 µL of each concentration of TEO and then placed on SDA plates. Fluconazole E-test strip was used as positive control and 1% Tween-20 were taken as negative control. The plates were left in laminar flow for 30 min to allow the diffusion of oil. The plates were incubated for 48 h at 37 °C. On completion of incubation, the diameter of the inhibition zone was recorded.

#### 2.4.2. Determination of Minimal Inhibitory (MIC) and Fungicidal Concentration (MFC)

The susceptibility of *C. albicans* against TEO was further tested by determining the MIC using a broth dilution assay [28]. *C. albicans* isolates were grown in the absence and presence of varying dilutions (0.3, 0.6, 1.25, 2.5, 5, 10, 20, and 40 µL/mL) of TEO in 10 mL SDB. One hundred µL of *C. albicans* isolate (~1.5 × 10^8^ CFU/mL) was taken as inoculum. The control group was not given any treatment and Tween-20 was used as the solvent control. The cultures were incubated for 48 h at 37 °C in a shaking incubator (200 rpm/min). The microbial growth was monitored calorimetrically at 540 nm. The lowest concentration of TEO that inhibited the fungal (no turbidity) was considered as MIC.

For the assessment of MFC, the treatment was given as mentioned in the MIC section [29]. Briefly, 100 µL of culture from each treatment dose was spread onto SDA plates and incubated at 37 °C for 48 h. The plates were observed for visible growth. The concentration at which 3 or lesser number of colonies were observed was taken as MFC.

#### 2.4.3. Effect of TEO on Budding of *C. albicans* Isolates

The effect of TEO on the budding of 10 *C. albicans* isolates was tested at their respective half inhibitory concentration (1/2 × MIC) [30]. Briefly, *C. albicans* isolates were cultured in the absence and presence of TEO for 24 h at 37 °C. The controls were also included. The fungal cells were observed under light microscope (40×) and one hundred cells were counted in each smear for the calculation of percentage of budding cells.

#### 2.4.4. Effect of TEO on Germ Tubes Formation of *C. albicans* Isolates

The effect of TEO on germ tubes formation of 10 strains of *C. albicans* was also tested at their respective 1/2×MICs. Briefly, the isolates were cultured at 1/2 × MIC of TEO for 3 h at 37 °C. Following incubation, 100 cells from each sample were counted using light microscope. The germ tubes were considered positive when germ tubes were seen arising from the yeast cells without a constriction at the point of their origin from the cells.

#### 2.4.5. Time Kill Curve Assay

The time kill assay of *C. albicans* was performed at its respective MFCs. The culture from log phase was taken and diluted to 0.5 McFarland turbidity (~1.5 × 10^8^ CFU/mL) and then 0.1 mL of each *C. albicans* isolate was used as inoculum [28]. The *C. albicans* isolates were cultured in the presence of the respective MFCs of TEO in 10 mL SDB. Fluconazole was used as a positive control and Tween-20 was taken as solvent control. Ten µL of culture were taken at varying time intervals (0, 4, 8, 12, 18, and 24 h) to make ten-fold serial dilution and 100 µL from each dilution was spread on SDA plates. The plates were incubated at 37 °C for 48 h and then the number of CFUs was counted.

#### 2.4.6. Effect of TEO on the *C. albicans* Ultrastructure by Scanning Electron Microscopic (SEM)

The inhibitory effect of TEO was further validated against *C. albicans* isolate no. 10. The isolate was grown in the absence and presence of TEO for 24 h at 37 °C. After incubation, the culture was taken on glass slides and fixed with 2.5% glutaraldehyde for 3 h. The glutaraldehyde was washed thrice with sodium cacodylate solution buffer and then post-fixed in osmium tetroxide for 1 h. The samples were then dehydrated using a graded ethanol series, 25%, 50%, 75%, and 100%, each for 10 min. The glass slides were coated with gold and observed under JSM-6380 LA field emission scanning electron microscope (JEOL, Musashino, Japan) at 20 KV.

## 3. Results

### 3.1. Candida *spp*. Isolates

In this study, 20 isolates of *Candida albicans* were selected from the total 120 clinical isolates of *Candida* spp. procured from the Prince Sultan Military Hospital, Riyadh, KSA. The obtained isolates were identified and characterized based on CHROMagar, germ tube development, carbohydrate metabolism, and RAPD-PCR technique.

#### 3.1.1. CHROMagar Based Identification of *Candida* spp.

Based on CHROMagar identification, the relative abundance of *C. albicans*, *C. glabrata*, *C. tropicalis*, and *C. krusei* was found to be 50.83, 34.1, 11.67, and 3.33%, respectively.

#### 3.1.2. Determination of Germ Tube Formation of *C. albicans*

**Figure 1:**
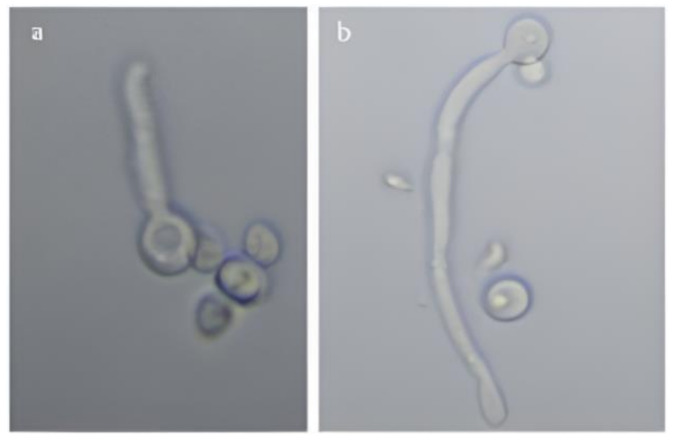
Formation of *C. albicans* Germ tube (**a**,**b**) in horse serum incubated for 3 h to form a germ tube at: 100× magnification.

From the average of 10 isolates, 20% of vaginal isolates showed germ tube formation while 26% of blood isolates exhibited this characteristic (Figure 1).

#### 3.1.3. Morphological Examination Using Microscopy

**Figure 2:**
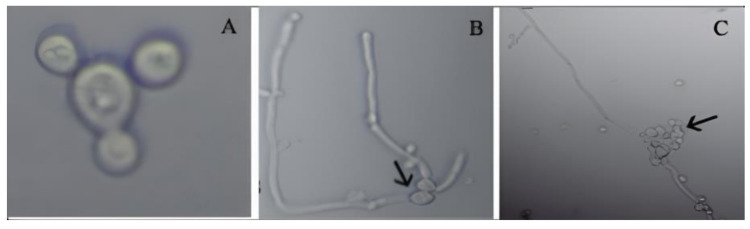
Various morphological forms of *C. albicans* at 100× magnification: (**A**) Budding, (**B**) Chlamydospores, (**C**) Blastospore. (**B**,**C**) Incubated in horse serum for 8 and 24 h, respectively.

The morphological examination of 50 isolates using microscopy showed that the fungal strains were unicellular, spherical to oval-shaped, budding yeast-like, and gram positive. *C. albicans* is dimorphic and changes its morphological form depending on environmental conditions. At room temperatures, the yeast form reproduces by budding, reaching a typical diameter of 8–10 µm. In some physiological conditions, *C. albicans* exhibits a hyphal form of growth called pseudohyphae, which are spherical and thick walled. This characteristic is usually produced in septulating cells. The blastospores are formed in grape-like clusters along the length of the hyphae (Figure 2). The microscopic examination showed that *C. albicans* was found to be the most frequently encountered species.

#### 3.1.4. API 20c Aux Based *C. albicans* Identification

In this study, 19 carbohydrate assimilation tests were performed using API 20C Aux system of yeast identification and the results were recorded after 1, 2, and 3 days. All 20 isolates were tested for carbohydrate metabolism as per the Analytical Profile Index provided in kit. Results were considered correct if results agree with the API reference identification and the profile was listed either as excellent, very good, or acceptable, as per the manufacturer instruction. The reaction of *C. albicans* on the API 20C Aux system ranged from 97% to 99%. A total of 30 isolates of *C. albicans* gave 99% reaction. API 20C Aux system-based identification of 20 isolates is presented in Table 1 and these 20 strains were selected for further studies. The isolates numbered from 1 to 10 were isolated from vagina samples and isolates designated from 11 to 20 were isolated from blood samples.

#### 3.1.5. Differentiation between *C. albicans* Isolates by RAPD-PCR Technique

**Figure 3:**
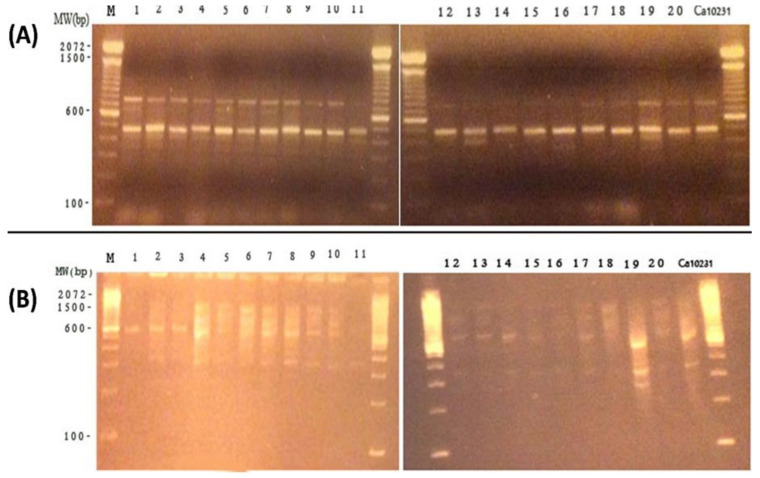
RAPD-PCR patterns generated by *C. albicans* isolates using (**A**) OPG 13 primer, (**B**) RSD 11 primer. Numbered lanes show patterns of the 20 *C. albicans* isolates. Ca10231: *C. albicans*102311. M:100 bp PCR DNA marker.

**Figure 4:**
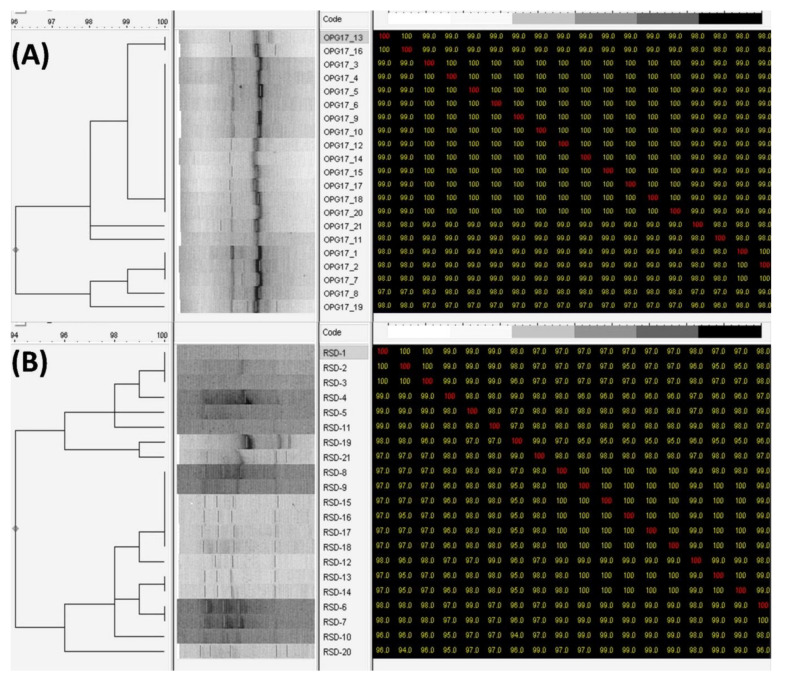
Dendrogram based RAPD-PCR data for *C. albicans* isolates from vaginal (1–10) and blood (11–20) in addition to identified isolate *C. albicans*102311: (**A**) primer OPG-17, (**B**) primer RSD11.

Out of the 4 primers used, two were selected, viz. OPG-17 and RSD-11, as these two primers presented reproducible DNA banding patterns for all *C. albicans* strains. The OPG-17 produced multiple banding patterns with fairly equal intensities and the same genetic profiles were obtained using OPG 17. On contrary, RSD 11 produced multiple banding patterns exhibiting fairly equal intensities eliciting 4 different genotypes. The RAPD profiles are shown in Figure 3. The two primers yielded RAPD profiles that ranged from 300 to 1800 base pairs (bp) for 20 clinical *C. albicans* isolates. In vaginal isolates (1–10), two bands of approximately 750 bp and 450 bp were found when amplified with OPG-17. The profile of blood isolates has only one common band with vaginal isolates at 450 bp. The value of similarity coefficient was found to be 98% and 96% using primer OPG-17 and RSD-11, respectively (Figure 4).

### 3.2. Extraction of Thyme Essential Oil (TEO)

The extraction of TEO was done using the Clevenger type apparatus following the standard procedure as described in the European Pharmacopoeia (Council of Europe). The yield of the TEO extracted from thyme was found to be 0.85%. Further, the detection of compounds present in TEO was performed using GC/MS analysis.

### 3.3. GC/MS Analysis of Thyme Essential Oil (TEO)

In GC/MS analysis, the identification was made by directly comparing the mass spectrum with NIST library. The GC/MS analysis of TEO showed the presence of 13 compounds (Table 2). The main constituents were found to be thymol (68.1%) followed by γ-terpinene (8.9%), cymol (7.7%), caryophyllene (1.1%), Linalool (1.4%). Many other phytocompounds were also detected but in lower amounts.

### 3.4. Antifungal Activity of Thyme Essential Oil (TEO) against C. albicans Isolates

The TEO was tested for its efficacy against the drug resistant isolates of *C. albicans*. The detailed findings are described below.

#### 3.4.1. Inhibitory Effect of TEO on *C. albicans* Isolates Using Disc Diffusion Assay

The preliminary antifungal effect of TEO was tested by disc diffusion assay. The TEO showed remarkable antifungal activity against all tested *C. albicans* isolates (Table 3 and Table 4). At the lowest tested concentration (2%), no inhibitory effect was found by disc diffusion assay. At 3% concentration, only one strain of *C. albicans* isolated from the vagina showed inhibition. At higher concentrations, a dose-dependent inhibitory effect was found against all *C. albicans* isolates. The average zone of inhibition against *C. albicans* vaginal isolates was recorded as 0.38 cm at 5% oil concentration. Likewise, average inhibition zone was 4.93 cm in the presence of 100% essential oil. A similar trend was observed against *C. albicans* blood isolates. Treatment with 5% and 10% TEO exhibited average inhibition zone as 1.39 and 1.79 cm, respectively. As evident from the data, 7 vaginal isolates and 8 blood isolates of *C. albicans* were resistant to fluconazole. Only a few strains (3 from vaginal isolates and 2 from blood isolates) were sensitive to fluconazole. Non-growth inhibition was recorded in the plates containing discs impregnated with Tween-20 (negative control). Overall, the comparison of results showed that essential thyme oil was more effective than fluconazole against the isolates.

#### 3.4.2. Determination of Minimal Inhibitory Concentration (MIC) and Fungicidal Concentration (MFC) of Thyme Essential Oil (TEO)

The inhibitory potential of TEO was further assessed by determining its MIC and MFC against all isolates of *C. albicans* (Table 5). A strong antifungal activity was recorded by the treatment of oil against *C. albicans* where 17 out of 20 isolates showed MIC as low as 0.6 µL/mL. At this concentration (0.6 µL/mL), the fungal growth was completely inhibited. The TEO were slightly lesser effective against isolates no. 4, 8, and 9. The fungicidal effect of TEO was also evaluated by determining the MFC. The MFCs was taken as the lowest concentration of TEO completely inhibiting the growth of *C. albicans* with fewer than 3 colonies of SDA plates. The presence of 1.25 µL/mL in culture medium was fungicidal to all strains of *C. albicans* isolated from blood. Most strains of *C. albicans* vaginal isolate showed MFC as 1.25 µL/mL. However, the TEO were fungistatic at higher concentration for 3 isolates (no. 4, 8, and 9).

#### 3.4.3. Effect of TEO on Budding of *C. albicans*

**Figure 5:**
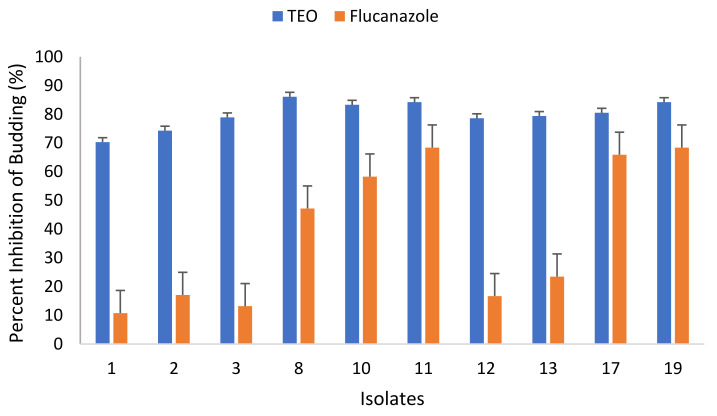
Percent inhibition of budding formation of *C. albicans* incubated at sub-inhibitory concentrations (1/2 × MIC) of the Thyme essential oil (TEO).

The effect of TEO used in this study on the budding of *C. albicans* isolates was also investigated and the results are represented in Figure 5. Results show that the TEO was more effective in inhibiting the bud formation compared to the positive control (i.e., fluconazole). The TEO interfered with the budding rate of all tested *C. albicans* isolates. The budding rate against all tested *C. albicans* isolates ranged from 13.9% to 29.7%. TEO was most effective against isolate no. 13 where only 13.9% budding was recorded. The lowest budding rate was observed in isolate no. 1. The budding rate in the presence of fluconazole ranged from 31.6% to 89.2%. Overall, the TEO was found to be effective in preventing the budding of all tested *C. albicans* isolates both from vagina and blood.

#### 3.4.4. Effect of TEO on Germ Tubes Formation of *C. albicans* Isolates

**Figure 6:**
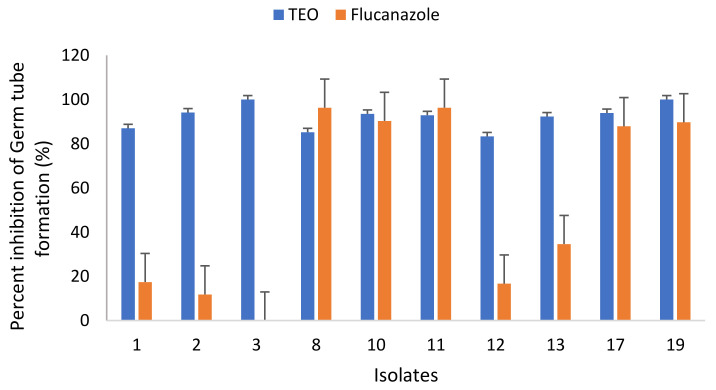
Percent inhibition of germ tube formation of *C. albicans* incubated at sub-inhibitory concentrations (1/2 × MIC) of the Thyme essential oil (TEO).

The effect of TEO in inhibiting the germ tube formation in *C. albicans* was also evaluated (Figure 6). The TEO was able to reduce the formation of germ tubes in all tested *C. albicans* isolates. For instance, the germ tube development in isolates no. 1 and 2 was found to be 13.0% and 5.9%, respectively. The germ tube formation in blood isolates of *C. albicans* were also reduced and only 7.1% and 16.7% germ tube formation in no. 11 and 12 was recorded. The results clearly show that germ tube formation was strongly inhibited by thyme oil at 1/2 × MIC. Moreover, the TEO were found to be effective in reducing the germ tube development of all tested *C. albicans* isolates, both from vagina and blood.

#### 3.4.5. Time-Kill Curves of *C. albicans* Isolates

**Figure 7:**
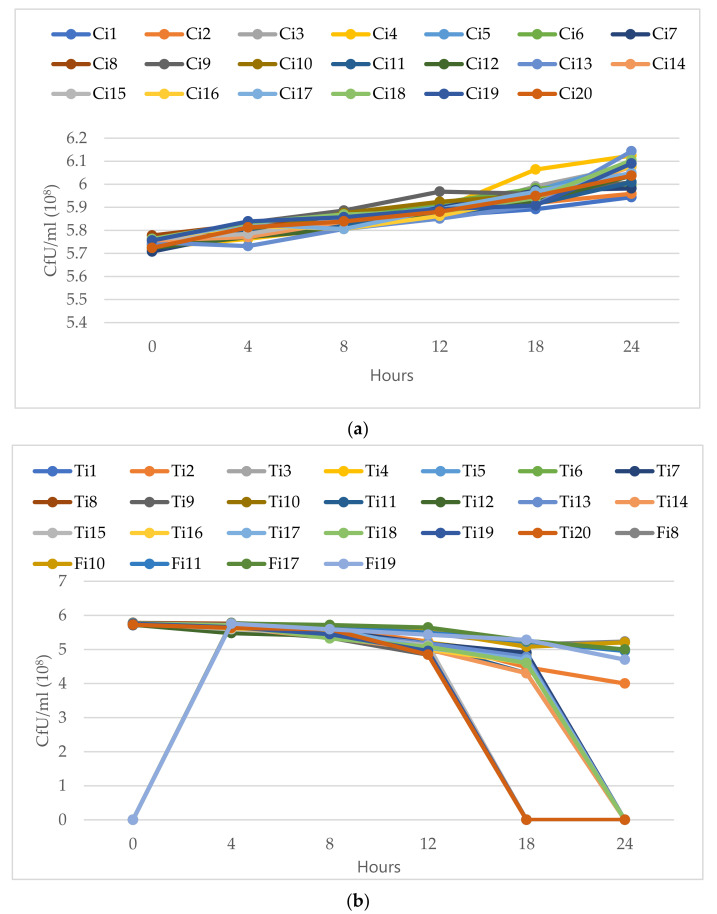
Time-kill of *C. albicans* on different times (0–24 h): (**a**) untreated (Control) isolates of *C. albicans* (Ci1–Ci20); (**b**) *C. albicans* isolates (Ti1–Ti20) treated with Minimum fungicidal concentration (MFC) dose of thyme essential oil (TEO) and *C. albicans* susceptible isolates (Fi8, Fi10, 1Fi1, Fi17, Fi19) treated with Fluconazole.

The fungicidal potential of TEO was examined using time kill assays. The findings showed that an excellent fungicidal effect was found at the respective MFCs. The *C. albicans* isolates were highly susceptible to tested oil at this concertation. The number of colonies for germinated cells was strongly reduced in all *C. albicans* isolates after 12 h of incubation. The complete fungicidal effect was observed at 18 h of incubation. For comparison, the fluconazole-resistant *C. albicans* isolates no. 8, 10, 11, 17 were also tested with fluconazole. The TEO were more effective than the fluconazole at lower doses (Figure 7). The *C. albicans* isolates treated with fluconazole showed regrowth from 18–24 h in isolates no. 8 and 10, which was not observed in presence of essential oil.

#### 3.4.6. Effect of TEO on the *C. albicans* Ultrastructure Detected by Scanning Electron Microscopy (SEM)

**Figure 8:**
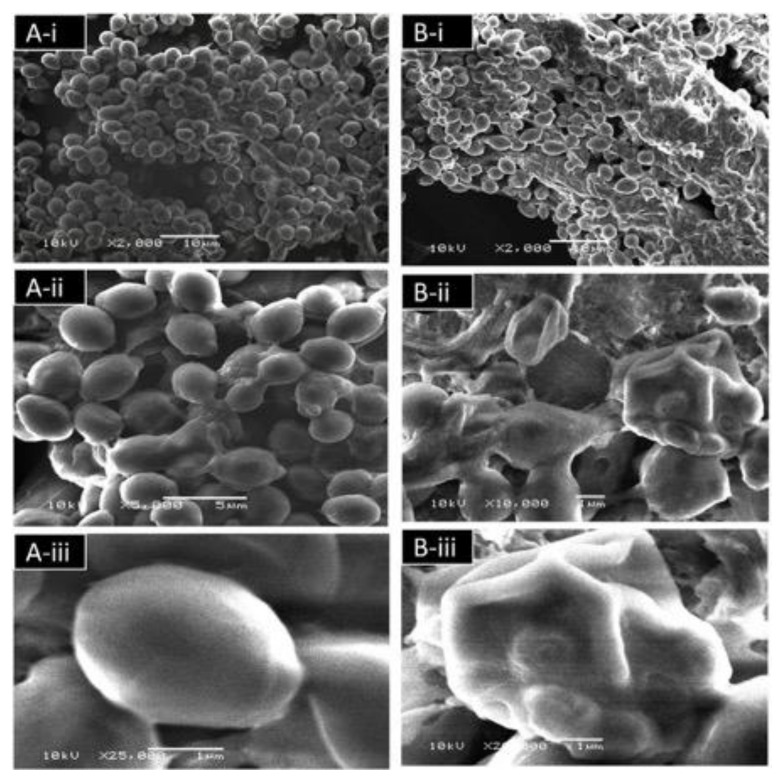
(**A**) Scanning electron micrographs showing *C. albicans* biofilm after 24 h without treatment at (**i**) 2000×, (**ii**) 5000×, and (**iii**) 25,000×. (**B**) Scanning electron micrographs showing effects on *C. albicans* biofilm after 24 h treatment with TEO at (**i**) 2000× (**ii**) 10,000×, and (**iii**) 25,000×.

In order to investigate the effect of the TEO on the ultrastructure of *C. albicans*, samples were examined by scanning electron microscopy (SEM). SEM analyses showed that untreated cells (control) exhibited a normal budding profile and had a typical structure with smooth wall (Figure 8A). Cells treated with TEO showed bumps and holes on the cell wall, which was not observed in control cells (Figure 8B).

## 4. Discussion

The *Candida* spp. infections have become common in hospitalized patients and their emergence is favored by immunosuppression. The *C. albicans* isolates were identified and characterized by employing several assays. The results prove that the most prevalent species in the collected samples were *C. albicans.* CHROMagar is differential culture medium which is routinely used for identification of clinical species of yeast. On this fungal culture medium, *C. albicans* produces distinctive green colonies, *C. glabrata* gives pink to purple colonies, *C. tropicalis* shows steel blue colonies, *C. krusei* shows rose coloured colonies with white edges [31,32,33]. Our findings corroborate an earlier report in which *C. albicans* was found to be the most common isolated species that caused candidemia at the Armed Forces Hospital, Riyadh, with 50.7% cases [1]. Moreover, it is also reported by several authors that the most frequently encountered species in *Candida* infections is *C. albicans* [32,34,35].

It has been noted that *C. albicans* fungemia is common in individuals suffering from respiratory infections or premature infants. On the other hand, patients with hepatic disorders or leukemia are vulnerable to *C. tropicalis* [36]. The clinical isolates of *C. albicans* formed germ tubes in horse serum when incubated at 37 °C. The formation of germ tubes within 2 h of incubation is the unique diagnostic characteristic of *C. albicans* that differentiates it from other fungi. As documented, up to 5% of *C. albicans* strains do not produce germ tube [37]. Usually, other yeasts do not form germ tubes within 3 h, neither *C. glabrata* nor *C. tropicalis*, although there are discrepancies as to which medium is best for germ tube production. For instance, some reports say that human serum is the best for the germ tube test [38,39]. However, other finding has suggested that trypticase soy broth is a better medium for testing germ tube production of *C. albicans* than human serum and horse serum [40]. Some studies that compared the performance of current chromogenic yeast identification have found that API 20C Aux was better than the other tests [24,41].

*C. albicans* is a pathogen that is primarily involved with systemic and superficial infections, affecting mainly chronic and immunocompromised patients; moreover, infection rates are rising as the number of such pateints rise [42]. The main advantage of RAPD-PCR over other methods of identification is that this technique is relatively faster and only takes a small amount of DNA. We intended to check whether these distinct primers could successfully amplify the DNA of isolates of *C. albicans*. The RSD-11 based genotypic differentiation of the isolates revealed the presence of four different genotypes in 20 *Candida* isolates. OPG-17, on the other hand, has the same genotype profile as the *Candida*. Our results deviate from a previous study that recorded four distinct genotypes in 14 *Candida* isolates when using OPG-17, whereas the same genotype profile was found with OPG-14 [43]. The pathogenicity of these isolates is probably owing to their genotypic features or because of the favorable growth environment they have within their hosts. The genotyping of *Candida* strains using PCR depends on the choice of primers [44]. The primers (OPG-17 and RSD-11) used in this work may be able to detect genomic variability among *C. albicans* isolates. Overall, the RAPD results showed that most patients exhibited varying and disparate genotypic patterns either within the same or different individuals.

In many countries, certain infectious diseases, including those caused by drug resistant pathogenic fungi, are treated with traditionally used herbal remedies. This had led the researcher to screen and search the potential new compounds for antifungal activities from natural sources, especially from ethnopharmacologically used plants. Owing to the toxicity of essential nature of oil, interests in essential oil with antifungal properties have increased. The essential oil yielded from the aerial part of *T. vulgaris* contains a high amount of thymol. The findings are in agreement with the earlier report which showed that thymol was a major component of thyme with a percentage ranging from 40–70% [45,46,47,48,49]. However, a high percentage of thymol is also reported in which thymol (84.45%) and carvacrol (46.62%) were the main components of *T. daenensis* and *T. vulgaris*, respectively [50]. Another finding has documented that a total of 33 compounds were identified in which thymol, cymol, γ-terpinene, caryophyllene, and α-terpinene were the major components in *T. vulgaris* [51]. Similarly, another study has found thymol (33.14%), carvacrol (19.59%), and linalool (16.00%) as the major components of thyme [52]. Variations in chemical composition of essential oils may be attributable to the various abiotic factors, geographic conditions, plant stage at the time of collection [53]. Despite the fact that essential oils’ antibacterial activity is mostly owing to their main components, the synergistic or antagonistic impact of a single chemical component in a small fraction of the essential oil composition has to be taken into account [9].

The antifungal activity of essential oils of natural origin is mainly owed to the bioactive phytocompounds that may be either acting individually or in synergy with other compounds [9]. Our results are in agreement with other studies where a remarkable antifungal activity of *Thymus* oil was found against *C. albicans* [54,55,56]. It is documented that thyme essential oil (TEO) exhibits strong antifungal activity on the growth of *C. albicans* with an inhibition zone of 56±1.5 mm [56]. Similarly, another finding has reported that the thyme oil was strongly fungistatic against *C. albicans* with zone of inhibition at 35 mm [57]. Moreover, essential oil obtained from *T. daenensis* was also found to produce good antifungal activity against *C. albicans* [50]. On the contrary, essential oil of thyme has also been found to exhibit weak antifungal activity against *C. albicans* isolates [58]. The variation in antifungal activity of essential oil of thyme not only depends on the strains of yeast tested, but also on the phytochemical composition of the plant which varies with the environment, season, and geographical locations. The GC/MS analysis revealed that thymol (73.1%) and γ-terpinene (9.9%) were the major components. Based on the available literature, it is inferred that the antifungal activity of essential oil mainly was due to the presence of thymol, carvacrol, and linalool [59,60].

At very low doses, the TEO proved to be fungistatic and fungicidal. This result is in concordance with a previous study which reported that thyme has the highest inhibitory activity against *C. albicans* [61]. Nevertheless, it is reasonable to speculate that the activity of TEO is owed to the presence of thymol and carvacrol since these compounds were detected in GC/MS. The relevance of phenolic hydroxyl groups for antimicrobial action has already been described [62,63,64]. *Thymus* species have been reported to be rich in phenol content and to have antifungal activity against several pathogenic fungi, including fluconazole resistant isolates [8,65,66]. Further, TEO was successful in inhibiting the bud formation. The result corroborates other studies which showed the reduction in bud formation of *C. albicans* by *O. gratissimum* essential oil. The probable reason for the lower budding rate may be the deleterious effect of the TEO on the pathogen’s cell wall, noting that the cell wall coherence is a key element in cell division [67]. Moreover, the TEO was found to be effective in reducing the germ tube development of all tested *C. albicans* isolates, both from vagina and blood. A similar finding has been reported earlier in which thymus oil significantly inhibited the germ tubes formation [66].

Overall, the TEO was found to be a more effective fungicide than fluconazole. This result is comparable with previous study that reported the *Candida* species treated with antifungal (nystatin) showed regrowth from 24–48 h, which was not observed with used TEO [67]. Moreover, the results are in concordance with another report which found that *C. albicans* was more susceptible to oregano oil and fungicidal effect was observed within 24 h [68]. It has been reported that linalool and eugenol killed 99.9% inoculums of *C. albicans* within 7.5 min [69]. Therefore, the killing of fungi by the active constituents may be attributed to fungicidal effects of the of essential oil.

SEM analysis further confirmed the damage caused by TEO to *C. albicans*. A similar result was reported earlier in which deformed cells with areas that resembled frustrated budding sites were observed in treated *C. albicans* with *O. gratissimum* (basil) oil [67]. Moreover, another study reported cells of *C. albicans* treated with oregano oil showed notable alterations in the cell wall [68]. A similar observation was also found using atomic force microscopy (AFM) where *C. albicans* cells showed major structural deformities at increasing thymol concentrations. A number of flattened cells with surface folds, cells with holes, and collapsed cells and ghosts were also seen [70]. Terpenes have been shown to modify the fluidity of the cell membrane, which affects permeability. This causes cell surface changes and abnormalities, which influence the pathogen’s adhesion and virulence. Thymol is an amphipathic monoterpene; therefore, it may have affected the cell membrane structure by generating imbalances and stresses in the cell membrane. Another study reported that thymol were more potent than eugenol in terms of its ability to interfere with the architecture of the *C. albicans* envelope [71].

## 5. Conclusions

Due to the global emergence and spread of antimicrobial resistance both in bacterial and fungal pathogens, the management of infectious diseases has become a daunting task. To overcome this problem, a lot of new strategies are being explored. One such strategy is to test the traditionally used medicinal plants, particularly essential oil, against the fungal pathogens. The phytochemical analysis revealed that thymol was the major component of hydro-distilled essential oil. Thyme essential oil (TEO) effectively inhibited the growth of *C. albicans* isolates. Moreover, the TEO was fungistatic and fungicidal at very low concentrations. The oil was also more potent in killing the fungal pathogens than fluconazole. This study provides experimental evidence for antifungal activity of essential of thyme that may be explored for the development of novel therapeutic agent, especially for the infection caused by *C. albicans*.

## Figures and Tables

**Table 1:** API 20C Aux results for *C. albicans*.

Isolate No.	Carbohydrate ^1^																		
	GLU	GLY	2KG	ARA	XYL	ADO	XLT	GAL	INO	SOR	MDG	NAG	CEL	LAC	MAL	SAC	TRE	MLZ	RAF
1	+	-	+	-	+	-	+	+	-	+	+	+	-	-	+	+	+	-	-
2	+	-	+	-	+	-	+	+	-	+	+	+	-	-	+	+	+	-	-
3	+	-	+	-	+	-	-	+	-	+	+	+	-	-	+	+	+	-	-
4	+	-	+	-	+	-	+	+	-	+	+	+	-	-	+	+	+	-	-
5	+	-	+	-	+	-	+	+	-	+	+	+	-	-	+	+	+	-	-
6	+	-	+	-	+	-	+	+	-	+	+	+	-	-	+	+	+	-	-
7	+	-	+	-	+	-	+	+	-	+	+	+	-	-	+	+	+	-	-
8	+	-	+	-	+	-	+	+	-	+	+	+	-	-	+	+	+	-	-
9	+	-	+	-	+	-	+	+	-	+	+	+	-	-	+	+	+	-	-
10	+	-	+	-	+	-	+	+	-	+	+	+	-	-	+	+	+	-	-
11	+	-	+	-	+	-	-	+	-	-	+	+	-	-	+	+	+	-	-
12	+	-	+	-	+	-	+	+	-	+	+	+	-	-	+	+	+	-	-
13	+	-	+	-	+	-	-	+	-	-	+	+	-	-	+	+	+	-	-
14	+	-	+	-	+	-	+	+	-	+	+	+	-	-	+	+	+	-	-
15	+	-	+	-	+	-	+	+	-	+	+	+	-	-	+	+	+	-	-
16	+	-	+	-	+	-	+	+	-	+	+	+	-	-	+	+	+	-	-
17	+	-	+	-	+	-	+	+	-	+	+	+	-	-	+	+	+	-	-
18	+	-	+	-	+	-	+	+	-	+	+	+	-	-	+	+	+	-	-
19	+	-	+	-	+	-	-	+	-	+	+	+	-	-	+	+	+	-	-
20	+	-	+	-	+	-	-	+	-	+	+	+	-	-	+	+	+	-	-

^1^ GLU: D-glucose; GLY: Glycerol; 2KG: Calcium 2-Keto-Gluconate; ARA: L-arabinose; XYL: D-xylose; ADO: Adonitol; XLT: Xylitol; GAL: D-galactose; INO: Inositol; SOR: D-sorbitol; MDG: Methyl-αd-Glucopyranoside; NAG: N-acetyl-glucosamine; CEL: D-cellobiose; LAC: D-lactose; MAL: D-maltose; SAC: D-saccharose (sucrose); TRE: D-trehalose; MLZ: D-melezitose; RAF: D-raffinose.

**Table 2:** Chemical composition of thyme essential oil (TEO) analysed by GC/MS.

No.	Compound Name	RI ^1^	%
1	α-Pinene	938	0.49
2	1-octen-3-ol	981	1.1
3	*β*-myrcene	995	0.4
4	3-octanol	998	0.3
5	α-Phellandrene	1008	0.2
6	α-Terpinene	1019	0.98
7	Cymol	1026	7.7
8	Limonene	1034	0.1
9	1,8-Cineole	1035	0.48
10	γ-Terpinene	1065	8.9
11	Terpinolene	1090	2.9
12	Linalool	1105	1.4
13	Borneol	1167	1
14	(-)4-trpineol	1177	0.6
15	γ-Terpineiol	1210	0.2
16	Thymol	1301	68.1
17	Carvacrol	1315	1.5
18	Isobornyl propionate	1379	0.27
19	Caryophyllene	1423	1.1
20	α-Humulene	1455	0.15
21	Germacrene-D	1482	0.12
22	γ-Cadinene	1520	0.18
23	Caryophyllene oxide	1584	0.9
24	α-Cadinol	1655	0.13
	Total		99.2
	Oil yield (%)		0.85

^1^ RI, Retention Indices.

**Table 3:** Zone of inhibition (cm) of *C. albicans* isolates from vagina at different concentrations of thyme essential oil (TEO).

TEO Concentration %	Isolates										
	1	2	3	4	5	6	7	8	9	10	Mean
2	0.00	0.00	0.00	0.00	0.00	0.00	0.00	0.00	0.00	0.00	0.00
3	0.77	0.00	0.00	0.00	0.00	0.00	0.00	0.00	0.00	0.00	0.17
5	0.90	0.00	0.87	1.03	0.83	0.00	0.00	0.73	0.70	1.20	0.38
10	1.03	1.93	2.10	2.13	1.07	0.87	0.87	1.10	1.03	1.77	0.80
15	2.30	2.10	2.30	2.27	1.17	1.07	0.90	2.13	1.63	2.00	1.14
20	2.50	2.47	2.40	2.77	1.17	1.60	2.17	2.37	2.00	2.47	1.69
25	3.50	2.80	3.30	3.63	2.50	2.03	2.57	3.53	2.17	2.80	2.45
30	3.90	3.00	3.77	3.80	4.00	3.30	2.80	4.03	3.20	3.83	3.27
100	5.27	5.13	5.17	5.37	5.43	5.07	4.83	5.27	5.30	5.07	4.93
Fluco	2.20	0.00	0.00	0.00	0.00	0.00	2.40	0.00	3.00	0.00	0.38
mean	2.0	1.7	1.6	2.0	2.0	1.4	1.4	1.1	1.3	2.0	
LSD 5%											
Concentration	0.201										
Isolates	0.181										
Conc × isolates	0.132										

**Table 4:** Zone of inhibition (cm) of *C. albicans* isolates from blood at different concentrations of thyme essential oil (TEO).

TEO Concentration %	Isolates										
	11	12	13	14	15	16	17	18	19	20	Mean
2	0.0	0.0	0.0	0.0	0.0	0.0	0.0	0.0	0.0	0.0	0.00
3	1.0	0.0	0.0	0.0	0.0	0.0	0.0	0.0	0.0	0.7	0.08
5	1.6	0.0	0.0	1.0	0.0	0.0	0.0	0.0	0.0	1.2	0.63
10	1.3	0.8	0.0	1.2	1.6	0.8	1.0	0.0	0.0	1.3	1.39
15	1.2	1.2	0.9	1.2	1.9	0.8	1.0	0.9	0.8	1.4	1.79
20	2.0	1.9	1.7	2.1	2.3	1.2	1.2	1.0	1.4	2.0	2.19
25	2.4	3.1	3.1	3.3	3.5	1.7	2.1	1.1	1.6	2.6	2.88
30	3.5	3.6	3.3	3.9	4.1	3.6	2.8	2.0	2.5	3.4	3.56
100%	4.9	5.1	5.1	5.2	4.8	4.8	4.7	4.6	5.1	5.1	5.19
Fluco	0.0	0.0	0.0	0.0	0.0	0.0	0.0	1.8	0.0	2.0	0.76
mean	2.24	1.94	2.21	2.33	1.80	1.55	1.57	2.13	1.78	2.13	
LSD 5%											
Concentration	0.0403										
Isolates	0.0419										
Conc × isolates	0.0967										

**Table 5:** Minimal Inhibitory Concentration (MIC) and Fungicidal Concentration (MFC) of the Thyme essential oil (TEO) against *C. albicans*.

Isolates	MIC		MFC	
	Thyme	Fluconazole	Thyme	Fluconazole
1	0.6	0	1.25	0
2	0.6	0	1.25	0
3	0.6	0	1.25	0
4	1.25	0	2.5	0
5	0.6	0	1.25	0
6	0.6	0	1.25	0
7	0.6	0	1.25	0
8	1.25	64	2.5	128
9	1.25	0	2.5	0
10	0.6	64	1.25	128
11	0.6	64	1.25	128
12	0.6	0	1.25	0
13	0.6	0	1.25	0
14	0.6	0	1.25	0
15	0.6	0	1.25	0
16	0.6	0	1.25	0
17	0.6	32	1.25	128
18	0.6	0	1.25	0
19	0.6	32	1.25	64
20	0.6	0	1.25	0

The sign “0” represents resistance to fluconazole.

## Data Availability

The data presented in this study are available in the article.

## References

[1] Al-Jasser A.M., Elkhizzi N.A. (2004). Distribution of Candida species among bloodstream isolates. Saudi Med. J..

[2] Badhman H. (2006). Variation in Growth of Candida albicans in Different Media. Ph.D. Thesis.

[3] Edwards J., Mandell G.L., Douglas R.G., Bennett J.E. (1995). Candida species. Principles and Practice of Infectious Diseases.

[4] Douglas L.J. (2003). Candida biofilms and their role in infection. Trends Microbiol..

[5] Eggimann P., Garbino J., Pittet D. (2003). Management of candidiasis Management of Candida species infections in critically ill patients. Lancet Infect. Dis..

[6] Al-Hedaithy S. (2002). Spectrum and proteinase production of yeasts causing vaginitis in Saudi Arabian women. Med. Sci. Monit..

[7] Kalemba D., Kunicka A. (2003). Antibacterial and Antifungal Properties of Essential Oils. Curr. Med. Chem..

[8] Dorman H.J.D., Deans S.G. (2000). Antimicrobial agents from plants: Antibacterial activity of plant volatile oils. J. Appl. Microbiol..

[9] Burt S. (2004). Essential oils: Their antibacterial properties and potential applications in foods—A review. Int. J. Food Microbiol..

[10] Bakkali F., Averbeck S., Averbeck D., Idaomar M. (2008). Biological effects of essential oils—A review. Food Chem. Toxicol..

[11] Khan A., Ahmad A., Manzoor N., Khan L.A. (2010). Antifungal Activities of Ocimum sanctum Essential Oil and its Lead Molecules. Nat. Prod. Commun..

[12] Ahmad A., Khan A., Manzoor N., Khan L.A. (2010). Evolution of ergosterol biosynthesis inhibitors as fungicidal against Candida. Microb. Pathog..

[13] Pinto E., Pina-Vaz C., Salgueiro L., Gonçalves M.J., de-Oliveira C.S., Cavaleiro C., Palmeira A., Rodrigues A., de-Oliveira M.J. (2006). Antifungal activity of the essential oil of Thymus pulegioides on Candida, Aspergillus and dermatophyte species. J. Med. Microbiol..

[14] Azzouz M., Bullerman L. (1982). Comparative Antimycotic Effects of Selected Herbs, Spices, Plant Components and Commercial Antifungal Agents1. J. Food Prot..

[15] Knobloch K., Pauli A., Iberl B., Weigand H., Weis N. (1989). Antibacterial and Antifungal Properties of Essential Oil Components. J. Essent. Oil Res..

[16] Ahmad I., Qais F., Samreen, Abulreesh H., Ahmad S., Rumbaugh K. (2019). Antibacterial Drug Discovery: Perspective Insights. Antibacterial Drug Discovery to Combat MDR.

[17] Edeoga H.O., Okwu D.E., Mbaebie B.O. (2005). Phytochemical constituents of some Nigerian medicinal plants. Afr. J. Biotechnol..

[18] Barreca S., La Bella S., Maggio A., Licata M., Buscemi S., Leto C., Pace A., Tuttolomondo T. (2021). Flavouring Extra-Virgin Olive Oil with Aromatic and Medicinal Plants Essential Oils Stabilizes Oleic Acid Composition during Photo-Oxidative Stress. Agriculture.

[19] Salehi B., Mishra A.P., Shukla I., Sharifi-Rad M., del Contreras M.M., Segura-Carretero A., Fathi H., Nasrabadi N.N., Kobarfard F., Sharifi-Rad J. (2018). Thymol, thyme, and other plant sources: Health and potential uses. Phyther. Res..

[20] Sharifi-Rad M., Varoni E.M., Iriti M., Martorell M., Setzer W.N., del Mar Contreras M., Salehi B., Soltani-Nejad A., Rajabi S., Tajbakhsh M. (2018). Carvacrol and human health: A comprehensive review. Phyther. Res..

[21] Kowalczyk A., Przychodna M., Sopata S., Bodalska A., Fecka I. (2020). Thymol and thyme essential oil—New insights into selected therapeutic applications. Molecules.

[22] Nadeem S.G., Hakim S.T., Kazmi S.U. (2010). Use of CHROMagar Candida for the presumptive identification of Candida species directly from clinical specimens in resource-limited settings. Libyan J. Med..

[23] Sheppard D.C., Locas M.-C., Restieri C., Laverdiere M. (2008). Utility of the Germ Tube Test for Direct Identification of Candida albicans from Positive Blood Culture Bottles. J. Clin. Microbiol..

[24] Gundes S., Gulenc S., Bingol R. (2001). Comparative performance of Fungichrom I, Candifast and API 20C Aux systems in the identification of clinically significant yeasts. J. Med. Microbiol..

[25] Scherer S., Stevens D. (1987). Application of DNA typing methods to epidemiology and taxonomy of Candida species. J. Clin. Microbiol..

[26] Abed K.M., Naife T.M. (2018). Extraction of Essential Oil from Iraqi Eucalyptus Camadulensis Leaves by Water Distillation Methods. IOP Conf. Ser. Mater. Sci. Eng..

[27] Khan M.S.A., Malik A., Ahmad I. (2012). Anti-candidal activity of essential oils alone and in combination with amphotericin B or fluconazole against multi-drug resistant isolates of Candida albicans. Med. Mycol..

[28] Jafri H., Banerjee G., Khan M.S.A., Ahmad I., Abulreesh H.H., Althubiani A.S. (2020). Synergistic interaction of eugenol and antimicrobial drugs in eradication of single and mixed biofilms of Candida albicans and Streptococcus mutans. AMB Express.

[29] Jafri H., Ahmad I. (2020). Thymus vulgaris essential oil and thymol inhibit biofilms and interact synergistically with antifungal drugs against drug resistant strains of Candida albicans and Candida tropicalis. J. Mycol. Med..

[30] Alshaikh N., Perveen K. (2017). Anti-candidal Activity and Chemical Composition of Essential Oil of Clove (Syzygium aromaticum). J. Essent. Oil Bear. Plants.

[31] Odds F., Bernaerts R. (1994). CHROMagar Candida, a new differential isolation medium for presumptive identification of clinically important Candida species. J. Clin. Microbiol..

[32] Yucesoy M., Marol S. (2003). Performance of CHROMAgar and BIGGY agar for identification of yeast species. Ann. Clin. Microbiol. Antimicrob..

[33] Sivakumar V.G., Shankar P., Nalina K., Menon T. (2009). Use of CHROMagar in the Differentiation of Common Species of Candida. Mycopathologia.

[34] Yera H., Poulain D., Lefebvre A., Camus D., Sendid B. (2004). Polymicrobial candidaemia revealed by peripheral blood smear and chromogenic medium. J. Clin. Pathol..

[35] Linares M.J., Charriel G., Solís F., Casal M. (2003). CHROMAgar Candida with fluconazole: Comparison with microdilution techniques. Enferm. Infecc. Microbiol. Clin..

[36] AlHedaithy S. (2003). The yeast species causing fungemia at a university hospital in Riyadh, Saudi Arabia, during a 10-year period. Das Hefespektrum der Fungamien an einem Universitatshospital in Riad, Saudi-Arabien, wahrend einer Zehnjahresperiode. Mycoses.

[37] Salkin I., Land G., Hurd N., Goldson P., McGinnis M. (1987). Evaluation of Yeast- Ident and Uni-Yeast-tek yeast identification systems. J. Clin. Microbiol..

[38] Makwana G., Gadhavi H., Sinha M. (2012). Comparison of germ tube production by Candida albicans in various media. NJIRM.

[39] Arora D.R., Saini S., Aparna, Gupta N. (2003). Evaluation of germ tube test in various media. Indian J. Pathol. Microbiol..

[40] Deorukhkar S., Saini S., Jadhav P. (2012). Evaluation of different media for germ tube production of Candida albicans and Candida dubliniensis. IJBAR.

[41] Schuffenecker I., Freydière A., Montclos H., Gille Y. (1993). Evaluation of four commercial systems for identification of medically important yeasts. Eur. J. Clin. Microbiol. Infect. Dis..

[42] Anwar K.P., Malik A., Subhan K.H. (2012). Profile of candidiasis in HIV infected patients. Iran. J. Microbiol..

[43] Costa C., Silva M., Souza L., ElAssal F., Ataíde F., Paula C. (2010). RAPD profile among Candida albicans isolates by using different primers. Rev. Patol. Trop..

[44] Dassanayake R., Samaranayake L. (2000). Characterization of the genetic diversity in superficial and systemic human isolates of Candida parapsilosis by randomly amplified polymorphic DNA (RAPD). APMIS.

[45] Khan A.A., Amjad M.S. (2019). Saboon GC-MS analysis and biological activities of Thymus vulgaris and Mentha arvensis essential oil. Turk. J. Biochem..

[46] Sefidkon F., Jamzad Z. (2005). Chemical composition of the essential oil of three Iranian Satureja species (S. mutica, S. macrantha and S. intermedia). Food Chem..

[47] Móricz Á., Ott P., Böszörményi A., Lemberkovics É., Mincsovics E., Tyihák E. (2012). Bioassay-Guided Isolation and Identification of Antimicrobial Compounds from Thyme Essential Oil by Means of Overpressured Layer Chromatography, Bioautography and GC–MS. Chromatographia.

[48] Moghtader M. (2012). Antifungal effects of the essential oil from Thymus vulgaris L. and comparison with synthetic thymol on Aspergillus niger. J. Yeast Fungal Res..

[49] Negahban M., Saeedfar S. (2015). Essential Oil Composition of Thymus vulgaris L.. Russ. J. Biol. Res..

[50] Behbahani M., Ghasemi Y., Khoshnoud M., Faridi P., Moradli G., Najafabady N. (2013). Volatile oil composition and antimicrobial activity of two Thymus species. Pharmacogn. J..

[51] Calín-Sánchez Á., Figiel A., Lech K., Szumny A., Carbonell-Barrachina Á.A. (2013). Effects of Drying Methods on the Composition of Thyme (Thymus vulgaris L.) Essential Oil. Dry. Technol..

[52] Omidbeygi M., Barzegar M., Hamidi Z., Naghdibadi H. (2007). Antifungal activity of thyme, summer savory and clove essential oils against Aspergillus flavus in liquid medium and tomato paste. Food Control.

[53] Gad H.A., Ayoub I.M., Wink M. (2019). Phytochemical profiling and seasonal variation of essential oils of three Callistemon species cultivated in Egypt. PLoS ONE.

[54] Pavel M., Alecu F. (2008). Antifungal activity of Thymus serpyllum essential oil against Candida albicans and Candida non-albicans clinical isolates: R2458. Clin. Microbiol. Infect..

[55] Naeini A., Khosravi A.R., Chitsaz M., Shokri H., Kamlnejad M. (2009). Anti- Candida albicans activity of some Iranian plants used in traditional medicine. J. Mycol. Med..

[56] Omran S., Esmailzadeh S. (2009). Comparison of anti-Candida activity of thyme, pennyroyal, and lemon essential oils versus antifungal drugs against Candida species. Jundishapur J. Microbiol..

[57] Ownagh A., Majdani R., Yaghobzadeh N., Nemati Z. (2008). Antifungal effects of Thyme oil on Candida albicans and Aspergillus fumigates. Iran. J. Vet. Med..

[58] Dalirsani Z., Adibpour M., Aghazadeh M., Amirchaghmaghi M., Mozafari P., Hamzei F. (2011). In vitro comparison of inhibitory activity of 10 plant extracts against Candida albicans. Aust. J. Basic Appl. Sci..

[59] Sivropoulou A., Papanikolaou E., Nikolaou C., Kokkini S., Lanaras T., Arsenakis M. (1996). Antimicrobial and Cytotoxic Activities of Origanum Essential Oils. J. Agric. Food Chem..

[60] Rota C., Carraminana J., Burillo J., Herrera A. (2004). In Vitro Antimicrobial Activity of Essential Oils from Aromatic Plants against Selected Foodborne Pathogens. J. Food Prot..

[61] Omran S., Esmaeilzadeh S., Rahmani Z. (2010). Laboratory study of anticandidal activity of thyme, pennyroyal and lemon essential oils by micro dilution method. Jundishapur J. Microbiol..

[62] Adam K., Sivropoulou A., Kokkini S., Lanaras T., Arsenakis M. (1998). Antifungal Activities of Origanum vulgare subsp. hirtum, Mentha spicata, Lavandula angustifolia, and Salvia fruticosa Essential Oils against Human Pathogenic Fungi. J. Agric. Food Chem..

[63] Aligiannis N., Kalpoutzakis E., Mitaku S., Chinou I.B. (2001). Composition and Antimicrobial Activity of the Essential Oils of Two Origanum Species. J. Agric. Food Chem..

[64] Nostro A., Blanco A.R., Cannatelli M.A., Enea V., Flamini G., Morelli I., Roccaro S.A., Alonzo V. (2004). Susceptibility of methicillin-resistant staphylococci to oregano essential oil, carvacrol and thymol. FEMS Microbiol. Lett..

[65] Nguefack J., Leth V., Zollo A.P.H., Mathur S.B. (2004). Evaluation of five essential oils from aromatic plants of Cameroon for controlling food spoilage and mycotoxin producing fungi. Int. J. Food Microbiol..

[66] Pina-Vaz C., Rodrigues G.A., Pinto E., de-Oliveira C.S., Tavares C., Salgueiro L., Cavaleiro C., Goncalves M., de-Oliveira M.J. (2004). Antifungal activity of Thymus oils and their major compounds. J. Eur. Acad. Dermatol. Venereol..

[67] Ueda-Nakamura T., Mendonça-Filho R.R., Morgado-Díaz J.A., Maza K.P., Filho B.P.D., Cortez A.G.D., Alviano D.S., do Rosa M.S.S., Lopes A.H.C.S., Alviano C.S. (2006). Antileishmanial activity of Eugenol-rich essential oil from Ocimum gratissimum. Parasitol. Int..

[68] Makki S., Olama Z., Holail H. (2012). Anti-fungal activity of plant oils against oral clinical isolates of Candida albicans in Lebanese community. Topclass J. Microbiol..

[69] Zore G.B., Thakre A.D., Jadhav S., Karuppayil S.M. (2011). Terpenoids inhibit Candida albicans growth by affecting membrane integrity and arrest of cell cycle. Phytomedicine.

[70] Braga P.C., Ricci D. (2011). Thymol-Induced Alterations in Candida albicans Imaged by Atomic Force Microscopy. Atomic Force Microscopy in Biomedical Research.

[71] Braga P., Sasso M., Culici M., Alfieri M. (2007). Eugenol and thymol, alone or in combination, induce morphological alterations in the envelope of Candida albicans. Fitoterapia.

